# Sphingomyelin synthase 2 regulates host protection conferred by the administration of *Cryptococcus neoformans* Δ*sgl*1 strain

**DOI:** 10.1016/j.jbc.2026.113330

**Published:** 2026-07-13

**Authors:** Neelu Singh, Tyler G. Normile, Deveney Dasilva, Nivea Pereira de Sa, Maurizio Del Poeta

**Affiliations:** 1 Department of Microbiology and Immunology, Stony Brook University, Stony Brook, New York, USA; 2 Division of Infectious Diseases, Stony Brook University, Stony Brook, New York, USA; 3 Veterans Affairs Medical Center, Northport, New York, USA

**Keywords:** *C. neoformans* Δ*sgl*1, IL-17A, phagocytosis, sphingomyelin synthase 2 (SMS2), sphingomyelin, toll like receptor (TLR2), γ/δ-T cells

## Abstract

Sphingolipids are fundamental components of the plasma membrane that regulate immune receptor organization and host responses to infection. Here, we identify sphingomyelin synthase 2 (SMS2) as a critical mediator of antifungal host defense against *Cryptococcus neoformans*. SMS2 deficiency reduces sphingomyelin and increases ceramide levels, altering membrane lipid composition and impairing receptor localization. This is associated with decreased plasma membrane toll-like receptor 2, attenuated signaling, and reduced IL-17A and IFNγ production by γδ T cells. Macrophages lacking SMS2 exhibit impaired phagocytosis, resulting in inefficient clearance of the attenuated *C. neoformans* Δ*sgl*1 strain. Consequently, SMS2-deficient mice fail to develop effective vaccine-induced protection and show increased susceptibility to secondary infection with WT *C. neoformans*. Mechanistically, bSMase-driven alterations in SM/ceramide balance are associated with disruption of membrane organization and reduced toll-like receptor 2 and cholera toxin B staining, suggesting that SMS2-dependent sphingomyelin regulation represents a key mechanism linking membrane lipid composition to innate immune signaling, phagocytosis, and antifungal host defense against *C. neoformans*.

*Cryptococcus neoformans* is an encapsulated opportunistic fungal pathogen that, after inhalation, disseminates to the central nervous system and causes life-threatening meningoencephalitis, particularly in HIV^+^ individuals (1). Current antifungal therapies—azoles, flucytosine, amphotericin B, and echinocandins—have major limitations: amphotericin B and flucytosine are toxic; flucytosine is not globally available; echinocandins have limited activity against *C. neoformans*; and azoles are constrained by drug–drug interactions and emerging resistance (2, 3). Consequently, mortality remains unacceptably high, underscoring the need for new therapeutic strategies (4, 5, 6).

In previous studies, we discovered that mice immunized with a *C. neoformans* Δ*sgl*1 mutant strain (accumulating ergosterol 3-β-glycoside) are completely protected from subsequent infection with virulent *C. neoformans* WT (7), even when depleted of CD4^+^ or CD8^+^ T cells, modeling the immunocompromised HIV^+^ host (8, 9). This protection depends on γ/δ-T cells, which produce high level of IFN-γ and IL-17A upon exposure to *C. neoformans* Δ*sgl*1 through toll-like receptor 2 (TLR2) signaling (10).

Sphingomyelin (SM) synthases catalyze the conversion of ceramide (CER) and phosphatidylcholine into SM and diacylglycerol, thereby regulating plasma membrane organization and signal transduction (11). Mammals express two isoforms, SMS1 and SMS2, with SMS2 particularly enriched in lung tissue and macrophages (12, 13, 14, 15, 16). In previous studies, we found that SMS2 is stimulated by *C. neoformans* (17) and is essential for macrophage-mediated control of the fungus (18). However, whether SMS2 regulates adaptive immune responses, including IFN-γ and IL-17A production upon *C. neoformans* exposure, remains unknown.

SM is a major structural component of lipid rafts, specialized membrane microdomains that organize immune receptors and signaling platforms. These lipid rafts transiently compartmentalize pattern recognition receptors, including toll-like receptors, to facilitate efficient signal transduction (19, 20, 21). Among them, TLR2 is a key receptor involved in sensing fungal components and initiating innate immune activation. However, whether SM availability regulates the anchoring of TLR2 at the plasma membrane has not been determined.

Here, we studied the effect of SMS2 deletion in the protective effect of *C. neoformans* Δ*sgl*1 through TLR2 signaling. We found that SMS2^−/−^ mice display reduced levels of SM in the bronchoalveolar lavage (BAL), in the lung tissue, and in serum. When infected with *C. neoformans* Δ*sgl*1 mutant, SMS2^−/−^ mice survived the infection but not capable to sustain a secondary infection by the WT *C. neoformans*. Deletion of SMS2 drastically diminishes the release of IFN-γ and IL-17A by γ/δ-T cells stimulated by either live or heat-killed *C. neoformans* Δ*sgl*1. The expression of TLR2 on the surface of γ/δ-T cells isolated from WT mice is increased by *C. neoformans* Δ*sgl*1 but drastically decreased in γ/δ-T cells isolated from SMS2^−/−^ mice. Finally, TLR2 expression is also diminished on the surface of macrophages by SM depletion, a membrane sphingolipid important for phagocytosis of *C. neoformans*. Together, these findings link SMS2-dependent SM homeostasis to TLR2 expression and the development of protective antifungal immunity.

## Results

### SMS2 deficiency decreases SM and increases ceramide levels in bronchoalveolar lavage, lung, and serum

**Figure 1:**
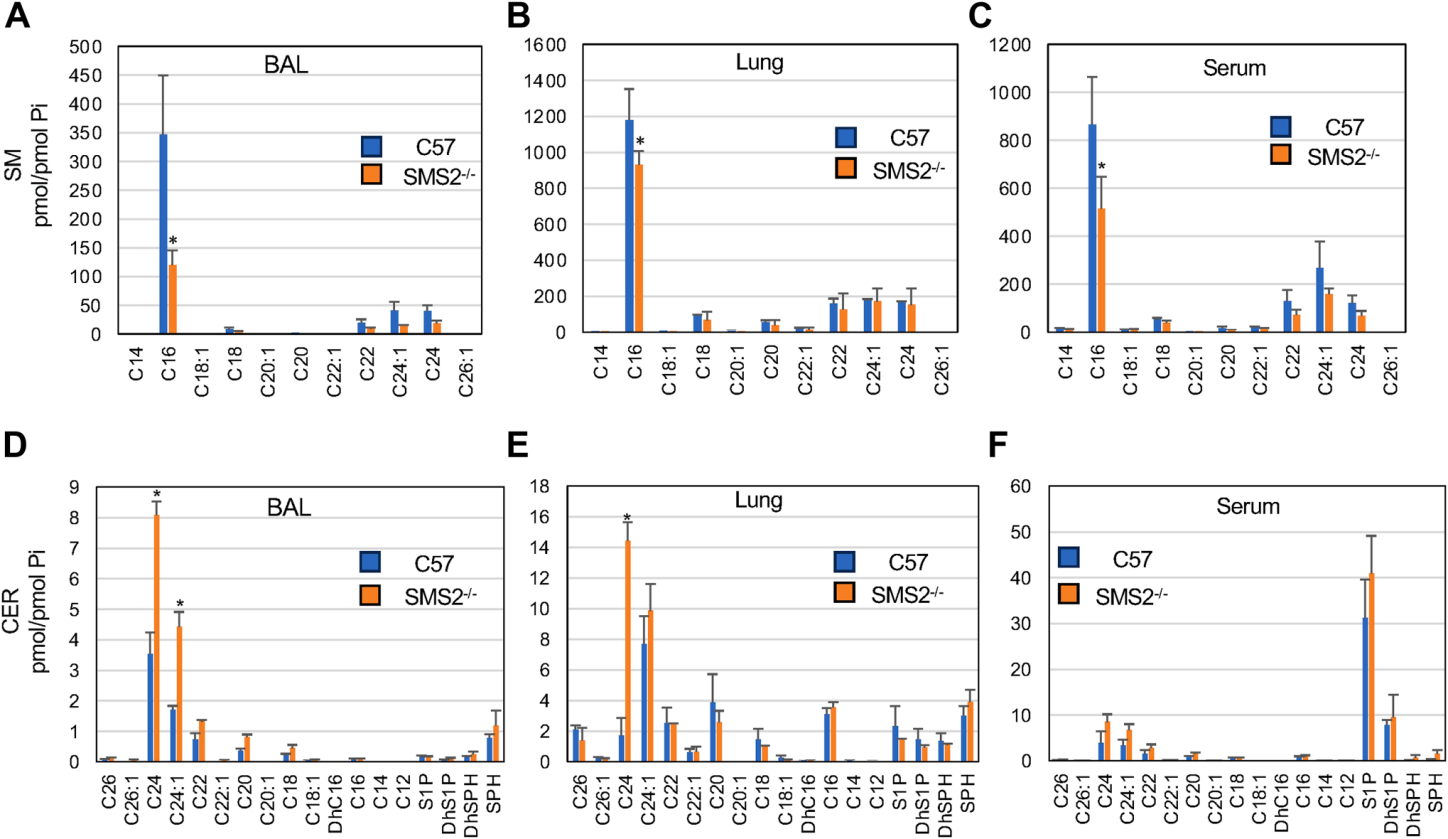
**Lipidomic analysis of sphingolipid species in WT (C57BL/6) and sphingomyelin synthase 2 KO (SMS2^−/−^) mice.***A, B* and *C*, SM levels in bronchoalveolar lavage fluid, lung homogenates, and serum collected from C57BL/6 and SMS2^−/−^ at 4 to 6 weeks of age. *D, E* and *F*, levels of CER, sphingosine 1 phosphate, dihydrosphingosine-1-phosphate, dihydrosphingosine , and sphingosine in bronchoalveolar lavage, homogenized lung tissue, and serum from C57BL/6 and SMS2^−/−^ at 4 to 6 weeks of age. Data are shown as mean ± SD (n = 6 mice per group; 3 females and 3 males). ∗*p* < 0.05, SMS2^−/−^*versus* C57BL/6, by one-way ANOVA with Tukey’s multiple comparisons test. BAL, bronchoalveolar lavage; CER, ceramide; SM, sphingomyelin; SMS2, sphingomyelin synthase 2.

SM and ceramide (CER) species were analyzed and quantified in bronchoalveolar lavage (BAL), lung homogenates, and serum by liquid chromatography-mass spectrometry. SMS2 deficiency resulted in a significant reduction of multiple SM species, including C16, C24, and C24:1 SM, with the most pronounced decrease observed in the BAL fluid (Fig. 1*A*) and more moderate reductions in lung tissue (Fig. 1*B*) and serum (Fig. 1*C*). Concomitantly, we observed an increase of C24 CER and C24:1 CER (Fig. 1, *D*–*F*).

In contrast, levels of other species of SM or CER or other sphingolipid intermediates, including sphingosine, dihydrosphingosine, sphingosine-1-phosphate (S1P), and dihydrosphingosien-1-phosphate (DhS1P), were not significantly altered in BAL, lung tissue, or serum from SMS2^−/−^ compared with WT C57BL/6 mice (Fig. 1, *D*–*F*). Together, these data indicate that SMS2 deletion selectively disrupts the SM–ceramide axis without broadly affecting downstream sphingoid base metabolites. These findings are consistent with previous reports showing that SMS2 deficiency reduces SM synthesis and leads to compensatory ceramide accumulation *in vivo* (11, 21, 22).

### SMS2 deletion abrogates vaccine-induced protection conferred by *C. neoformans* Δ*sgl*1

Because SM has been implicated in macrophage phagocytosis of *C. neoformans* and is enriched in BAL (18), we first assessed the impact of SMS2 deficiency on susceptibility to *C. neoformans* infection. Initially, we infected C57BL/6 or SMS2^−/−^ mice with *C. neoformans* WT H99 and compared mice survival. We found that the average survival of C57BL/6 mice was 21 ± 3.8 days, whereas the average survival of SMS2^−/−^ mice was 16 ± 2.6, which is significantly lower (data not shown). In contrast, infection with the attenuated Δ*sgl*1 strain was non-lethal in both C57BL/6 or SMS2^−/−^ mice, with all animals surviving the 30-days of observation period without any signs of disease (data not shown), indicating that SMS2 deficiency does not impair control of primary infection with the attenuated strain.

**Figure 2:**
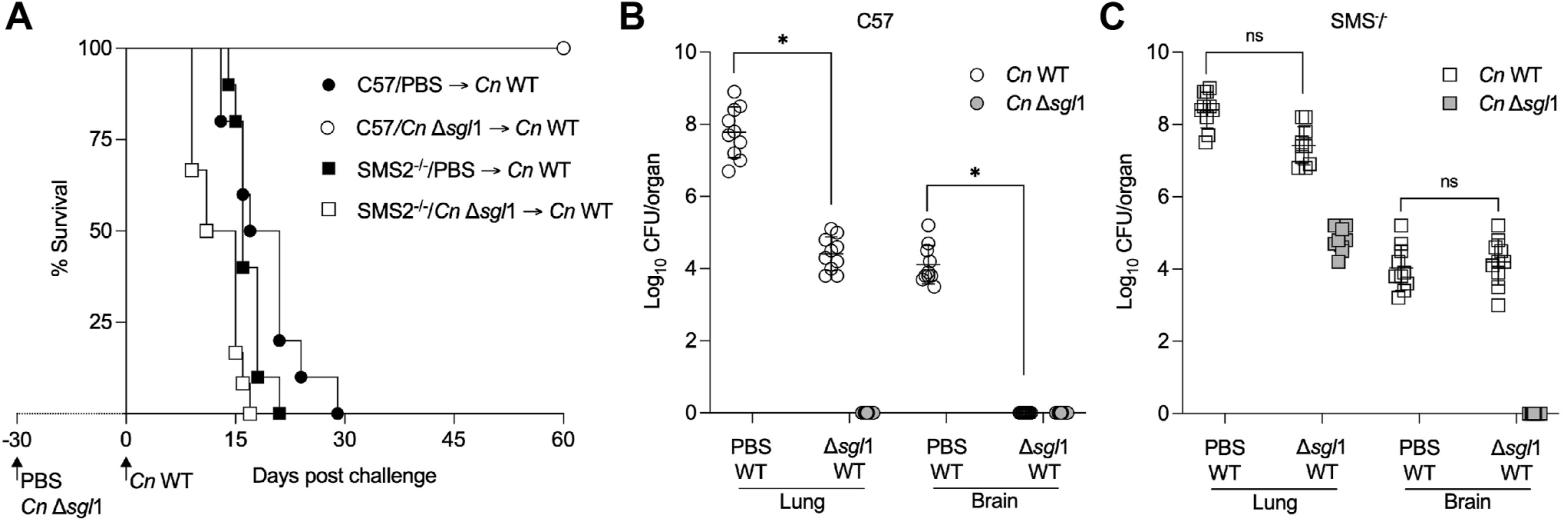
**Effect of SMS2^−/−^ deletion on vaccination with *C. neoformans* Δ*sgl*1.***A*, percentage Survival of WT C57BL/6 (C57) and SMS2^−/−^ mice treated with either PBS or *Cn* Δ*sgl*1 (n = 10 mice per group) and then challenged after 30 days with *C. neoformans* (Cn) WT H99 (*Cn* WT). ∗*p* < 0.05, C57/*Cn* Δ*sgl*1 *versus* any of the other groups by Kruskal-Wallis test. *B*, tissue fungal burden was calculated by the CFU in the Lung and Brain of C57BL/6 mice. For PBS-treated mice, CFU was calculated at the time-of-death, whereas for *Cn* Δ*sgl*1-treated mice, CFU was calculated at the end-of-study (60 days). n = 10 mice per group. Data represent mean ± SD. ∗*p* < 0.05 by one-way ANOVA with Tukey’s multiple comparisons test. *C*, tissue fungal burden by CFU in the lung and brain of SMS2^−/−^ mice. For all SMS2^−/−^ mice, CFU was calculated at the time-of-death (n = 10 mice per group). Data represent mean ± SD. ns, not significant, as determined by one-way ANOVA with Tukey’s multiple comparisons test. CFU, colony forming unit; SMS2, sphingomyelin synthase 2.

We next examined whether SMS2 is required for the protective immunity induced by Δ*sgl*1 vaccination. A group of C57BL/6 or SMS2^−/−^ mice were vaccinated with either live 5 × 10^5^ *C. neoformans* Δ*sgl*1 cells or received sterile phosphate buffered saline (PBS) and, after 30 days, challenged with 5 × 10^5^ *C. neoformans* WT H99 cells (Fig. 2*A*). As expected, vaccinated C57BL/6 mice were fully protected, with 100% survival through 60 days post challenge, whereas mice receiving PBS succumbed to infection. Interestingly, SMS2^−/−^ mice failed to develop vaccine-induced protection and displayed survival curves comparable to non-vaccinated controls mice receiving PBS (Fig. 2*A*).

Consistent with the survival data, vaccinated C57BL/6 mice exhibited low pulmonary fungal burden (∼10^4^ CFU of WT *C. neoformans* H99) with no detectable dissemination to the brain at the 60-days endpoint (Fig. 2*B*). No Δ*sgl*1 colonies were detected at the endpoint in these mice, neither in lung nor in brain (Fig. 2*B*). In contrast, SMS2^−/−^ vaccinated mice showed significantly higher fungal burdens (∼10^8^ and 10^4^ CFU) in both lungs and brains following challenge with *C. neoformans* H99 (Fig. 2*C*). Notably, Δ*sgl*1 colonies were also recovered from the lungs of SMS2^−/−^ mice at the time of analysis (Fig. 2*C*), suggesting impaired clearance or persistence of the Δ*sgl*1 strain in the absence of SMS2.

### Effect of SMS2 deletion on clearance of *C. neoformans* Δ*sgl1* strain and sustained protective immunity following extended vaccination

**Figure 3:**
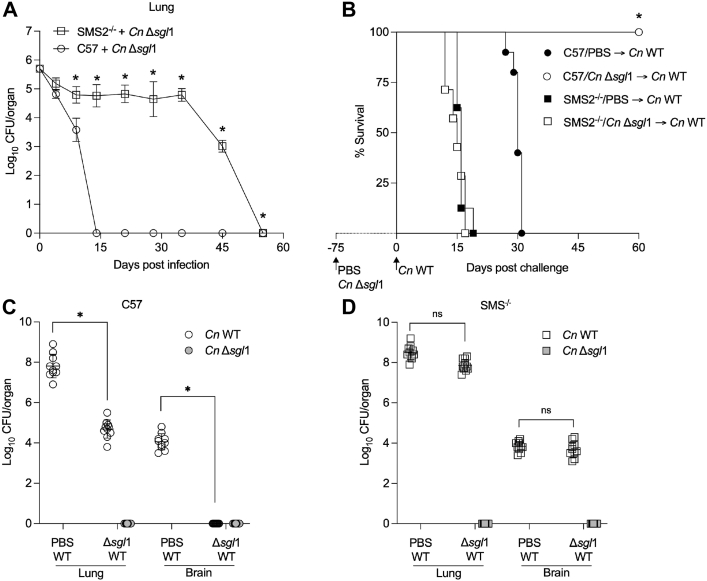
**Effect of SMS2^−/−^ deficiency on *Cryptococcus neoformans* Δ*sgl*1 clearance and extended vaccination efficacy.***A*, clearance of *C. neoformans* Δ*sgl*1 *(Cn* Δ*sgl*1) in WT C57BL/6 (C57) mice and SMS2^−/−^ mice was evaluated by lung colony forming unit (CFU) measured during a period of 60 days (n = 4 mice per time point). Data are presented as mean ± SD. ∗*p* < 0.05 SMS2^−/−^*versus* C57, by one-way ANOVA with Tukey’s multiple comparisons test. *B*, survival of C57BL/6 and SMS2^−/−^ mice following extended vaccination with PBS or *Cn* Δ*sgl*1 and subsequent challenge with virulent *Cn* H99 (WT) at 75 days post-vaccination (n = 10 mice per group). *C*, fungal burden (CFU) in lung and brain of C57BL/6 mice. For PBS-treated mice, CFU was calculated at the time-of-death, whereas for *Cn* Δ*sgl*1-vaccinated mice, CFU was calculated at the end-of-study (60 days post challenge; n = 10 mice per group). Data are presented as mean ± SD. ∗*p* < 0.05, by one-way ANOVA with Tukey’s multiple comparisons test. *D*, lung and brain fungal burden by CFU in SMS2^−/−^ mice (n = 10 mice per group). For all SMS2^−/−^ mice, CFU was calculated at the time-of-death. Data are presented as mean ± SD. ns, not significant, by one-way ANOVA with Tukey’s multiple comparisons test. SMS2, sphingomyelin synthase 2.

To determine whether SMS2^−/−^ mice are able to efficiently clear *C. neoformans* Δ*sgl*1 cells; we performed a time-course analysis of fungal burden in lung tissue over 60 days. C57BL/6 or SMS2^−/−^ mice were infected with *C. neoformans* Δ*sgl*1, and lungs were harvested at the indicated time points for CFU quantification. C57BL/6 mice cleared Δ*sgl1* from the lungs within 14 days, as expected, whereas SMS2^−/−^ mice required significantly longer with clearance occurring only by approximately 57 days (Fig. 3*A*).

Based on this delayed clearance, we repeated the vaccination experiment, extending the interval to 75 days after immunization with live *C. neoformans* Δ*sgl*1 in C57BL/6 or SMS2^−/−^ mice prior to challenge with WT *C. neoformans* H99 (Fig. 3*B*). As shown, vaccinated C57BL/6 mice were fully protected and survived the challenge, whereas vaccinated SMS2^−/−^ mice succumbed to infection, surviving less than 20 days post-challenge, similarly to the SMS2^−/−^ mice that received PBS instead of vaccine. In contrast, C57BL/6 mice that received PBS instead of the vaccine died within approximately 30 days after challenge (Fig. 3*B*).

Fungal burden analysis from the extended vaccination study revealed that vaccinated C57BL/6 mice effectively controlled WT H99 growth in the lungs (∼10^4^ CFU/organ), whereas PBS-treated controls exhibited high pulmonary fungal burden (∼10^8^ CFU/organ). No dissemination to the brain was detected in vaccinated mice, while PBS-treated animals showed significant brain infection (∼10^4^ CFU/organ) (Fig. 3*C*). In contrast, vaccinated SMS2^−/−^ mice exhibited uncontrolled fungal growth in the lungs (∼10^8^ CFU/organ) with subsequent dissemination to the brain (∼10^4^ CFU/organ), comparable to SMS2^−/−^ mice receiving PBS (Fig. 3*D*). As expected, Δ*sgl1* organisms were not detectable in the lungs of vaccinated C57BL/6 or SMS2^−/−^ mice at the endpoint (Fig. 3*D*).

Sphingolipid biosynthesis has been implicated in phagocytic cup formation and actin remodeling required for efficient pathogen uptake; thus, defects in this pathway may impair pathogen clearance and promote persistence (23). Collectively, these findings demonstrate that SMS2 is critical for effective clearance of the *C. neoformans* Δ*sgl*1 strain and for the development of strong protective immunity against a secondary infection.

### Deletion of SMS2 results in attenuated release of IL-17A and IFNγ by γδ T cell stimulated by live or heat-killed *C. neoformans* Δ*sgl*1

**Figure 4:**
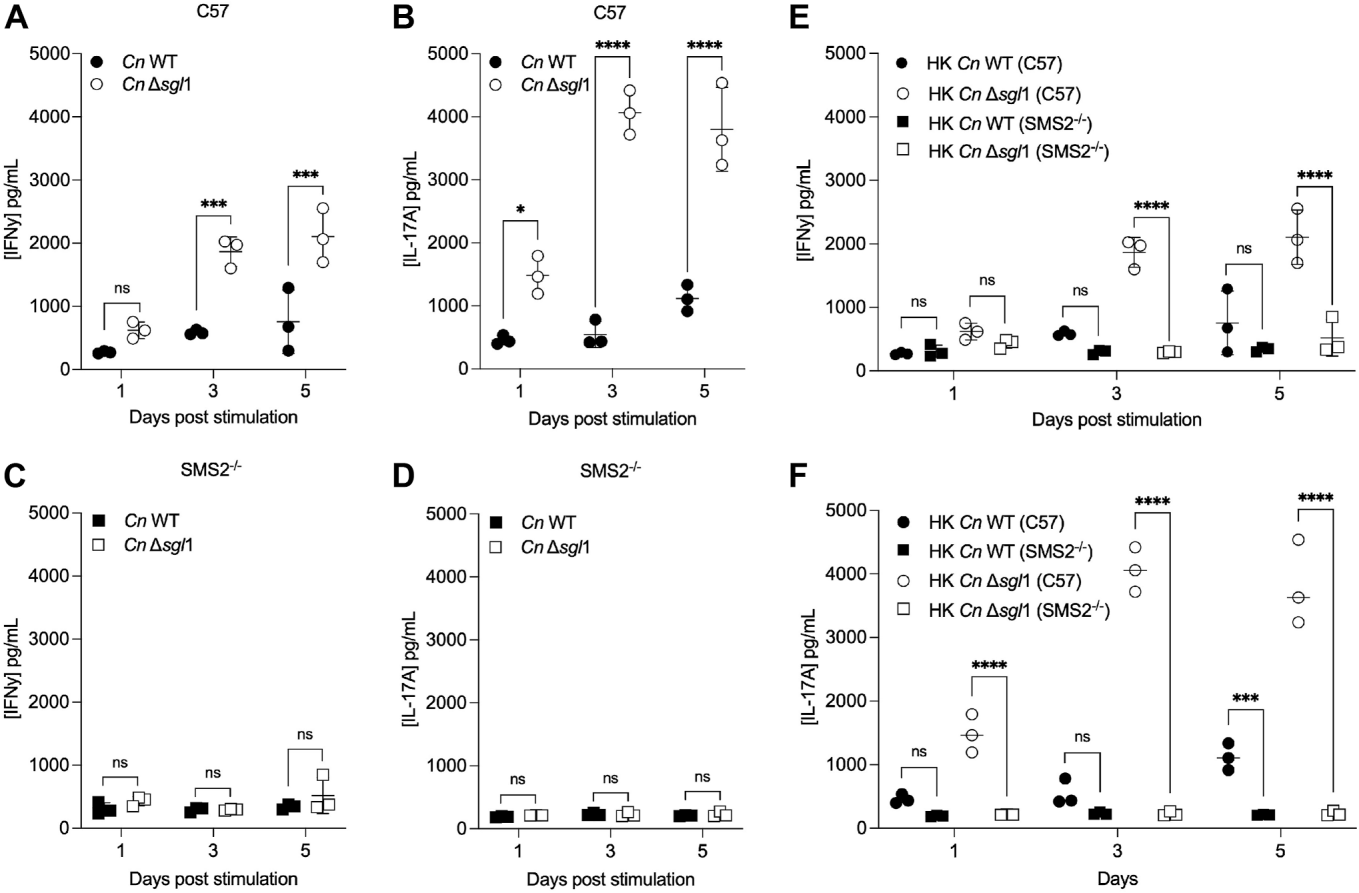
**Deletion of sphingomyelin synthase 2 (SMS2^−/−^) diminishes the release of IL-17A and IFNγ by γδ-T cells stimulated with live or heat-killed *C. neoformans* Δ*sgl*1.***A* and *B*, γδ-T cells isolated from C57BL/6 (C57) mice showed a significant time-dependent increase in IFNγ (*A*) or IL-17A (*B*) production upon stimulation with live *Cn* Δ*sgl*1, compared to live *Cn* WT (n = 3 mice per group at each time point). *C* and *D*, in contrast, γδ-T cells isolated from SMS2^−/−^ mice show no significant IFNγ (*C*) or IL-17A (*D*) stimulation by either live *Cn* Δ*sgl*1or live *Cn* WT (n = 3 mice per group at each time point). Data represent mean ± SD. ∗∗∗*p* < 0.001, ∗∗∗∗*p* < 0.0001, by one-way ANOVA with Tukey’s multiple comparisons test. *E* and *F*, IFNγ (*E*) and IL-17A (*F*) production stimulated by heat-killed *Cn* WT or *Cn* Δ*sgl*1 by γδ-T cells isolated from either C57 of SMS2^−/−^ mice (n = 3 mice per group at each time point). Data represent mean ± SD. ∗∗∗*p* < 0.001, ∗∗∗∗*p* < 0.0001, by one-way ANOVA with Tukey’s multiple comparisons test. SMS2, sphingomyelin synthase 2.

Our previous study showed that *C. neoformans* Δ*sgl*1 exert its protective effect through the activation of γδ-T cells and their release of IL-17A and IFNγ (10). Thus, we investigated the release of IL-17A and IFNγ cytokine by γδ-T cells isolated from C57BL/6 or SMS2^−/−^ mice following the stimulation with either live or heat-killed *C. neoformans* Δ*sgl*1 or WT *C. neoformans* H99 cells (WT). As previously observed (10), when using γδ-T cells from WT C57BL/6 mouse, we found a robust and time-dependent increase in IL-17A and IFNγ production following incubation with live Δ*sgl*1 cells with significantly (∗∗∗*p <* 0.001, *∗∗∗∗p* < 0.0001) higher levels observed at days 3 and 5 (Fig. 4, *A* and *B*), which was significantly higher than γδ-T cells exposed to WT (Fig. 4, *A* and B). In contrast, γδ-T cells isolated from SMS2^−/−^ mice failed to produce a robust and a time-dependent release of IL-17A and IFNγ cytokines when stimulated by either Δ*sgl*1 or WT cells (Fig. 4, *C* and *D*).

A similar trend was observed in γδ-T cells from C57BL/6 mice incubated with heat-killed (HK) *C. neoformans* Δ*sgl*1, which still effectively induced IL-17A and IFNγ production significantly (∗∗∗∗*p* < 0.0001) at day 3 and 5 compared to HK WT (Fig. 4*E*). However, the release of IL-17A or IFNγ by γδ-T cells isolated from SMS2^−/−^ mice remains unaltered upon exposure to HK Δ*sgl*1 compared to WT cells with no time-dependent effect (Fig. 4, *E* and *F*). These findings suggest that SMS2 plays a critical role in mediating the release of IL-17A and IFNγ in γδ-T cells stimulated by Δ*sgl*1. However, effective immune responses rely on the coordinated interplay between innate and adaptive immunity, with γδ T cells and TLRs acting as critical mediators bridging these two arms of the immune system.

### SMS2 deficiency impairs TLR2 localization at the plasma membrane in γδ T cells

TLRs are type I transmembrane proteins that recognize conserved molecular patterns derived from various pathogens, including fungi, and are major contributors to the mammalian innate immune system (24). Recent studies have shown that both human and murine γδ T cells express functional TLRs, including TLR2, supporting their role in mediating early immune responses to bacterial and other microbial infections (25). Upon activation, TLRs initiate signaling cascades that promote the release of pro-inflammatory mediators, including cytokines (26).

Because the localization of TLRs at the plasma membrane is normally required for the production of proinflammatory cytokines in response to extracellular pathogens (27, 28), we investigated whether deletion of SMS2 would affect the localization of TLR2 on the surface of γδ-T cells in the presence or absence of *C. neoformans* Δ*sgl*1.

**Figure 5:**
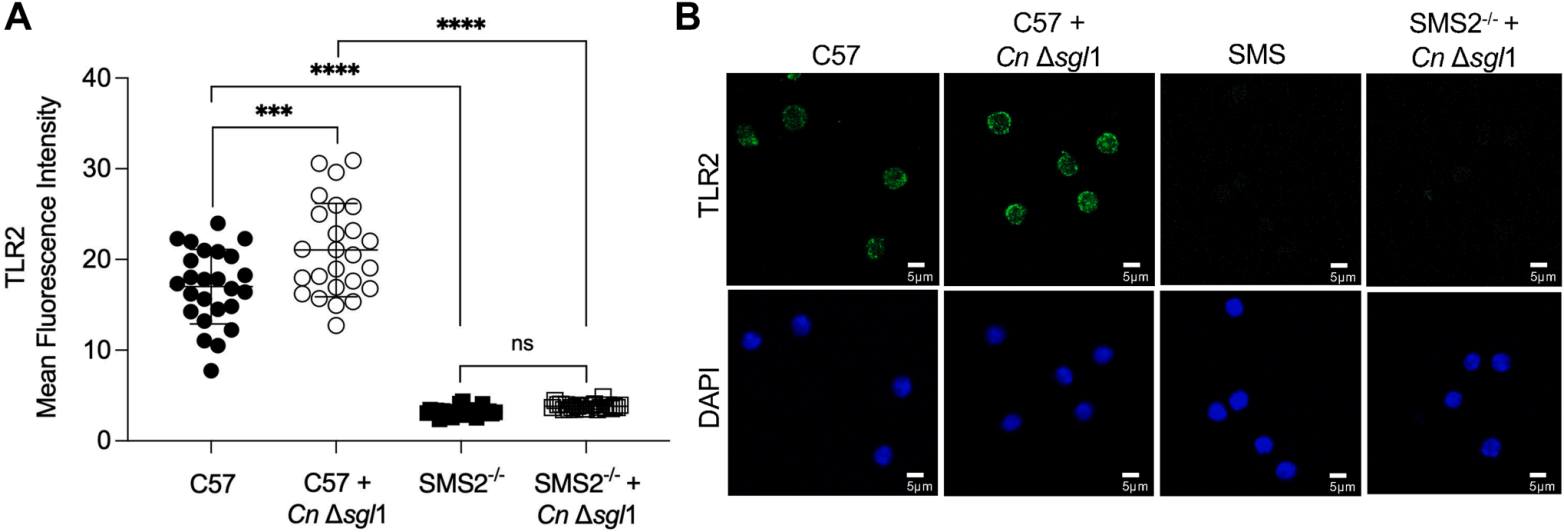
**Deletion of SMS2 abrogates stimulation of TLR2 expression in γδ-T cells by *C. neoformans* Δ*sgl*1.** γδT cells were isolated from WT C57BL/6 (C57) mice or from full KO mice in which was deleted (SMS2^−/−^). *A*, *Cn* Δ*sgl*1 stimulates TLR2 expression on the surface of γδ-T cells in C57 mice but not in SMS2^−/−^ mice. Deletion of SMS2 dramatically decreases the expression of TLR2 on the surface of unstimulated γδ-T cells and addition of *Cn* Δ*sgl*1 did not increase TLR2 expression. TLR2 expression was assessed by immunofluorescence staining (MFI) and analyzed using high-resolution confocal microscopy. Each *data point* represents a single γδ T cell, based on 8 to 10 micrographs per experiment across two independent experiments (n = 3 mice per group per experiment). Data are presented as mean ± SD. Statistical significance was determined by one-way ANOVA with Tukey’s multiple comparisons test. ∗∗∗*p* < 0.001; ∗∗∗∗*p* < 0.0001; ns, not significant. *B*, representative confocal images of the graph illustrated in 5A. TLR2 is shown in *green*, and nuclei are counterstained with DAPI (*blue*). The scale bar represents = 5 μm. MFI, Mean Fluorescence Intensity; SMS2, sphingomyelin synthase 2; TLR2, toll-like receptor 2.

We found a dramatic reduction in TLR2 fluorescent signal in γδ-T cells isolated from SMS2^−/−^ compared to γδ-T cells isolated from C57BL/6 mice (Fig. 5, *A* and *B*). Interestingly, incubation with *C. neoformans* Δ*sgl*1 increases TLR2 fluorescent signal at the plasma membrane of C57BL/6 γδ-T cells but not SMS2^−/−^ γδ-T cells (Fig. 5, *A* and *B*). These results suggest a key role for SMS2, perhaps through SM, in anchoring TLR2 on the surface of these innate cells.

### Depletion of SM decreases TLR2 localization at the plasma membrane

Because we found that TLR2 localization depends on SMS2 and that SMS2 is mainly localized at the plasma membrane, we investigated whether a depletion of SM in the outer leaflet of the plasma membrane would affect the localization of TLR2. For this set of experiments, we used MHS macrophage cell line.

**Figure 6:**
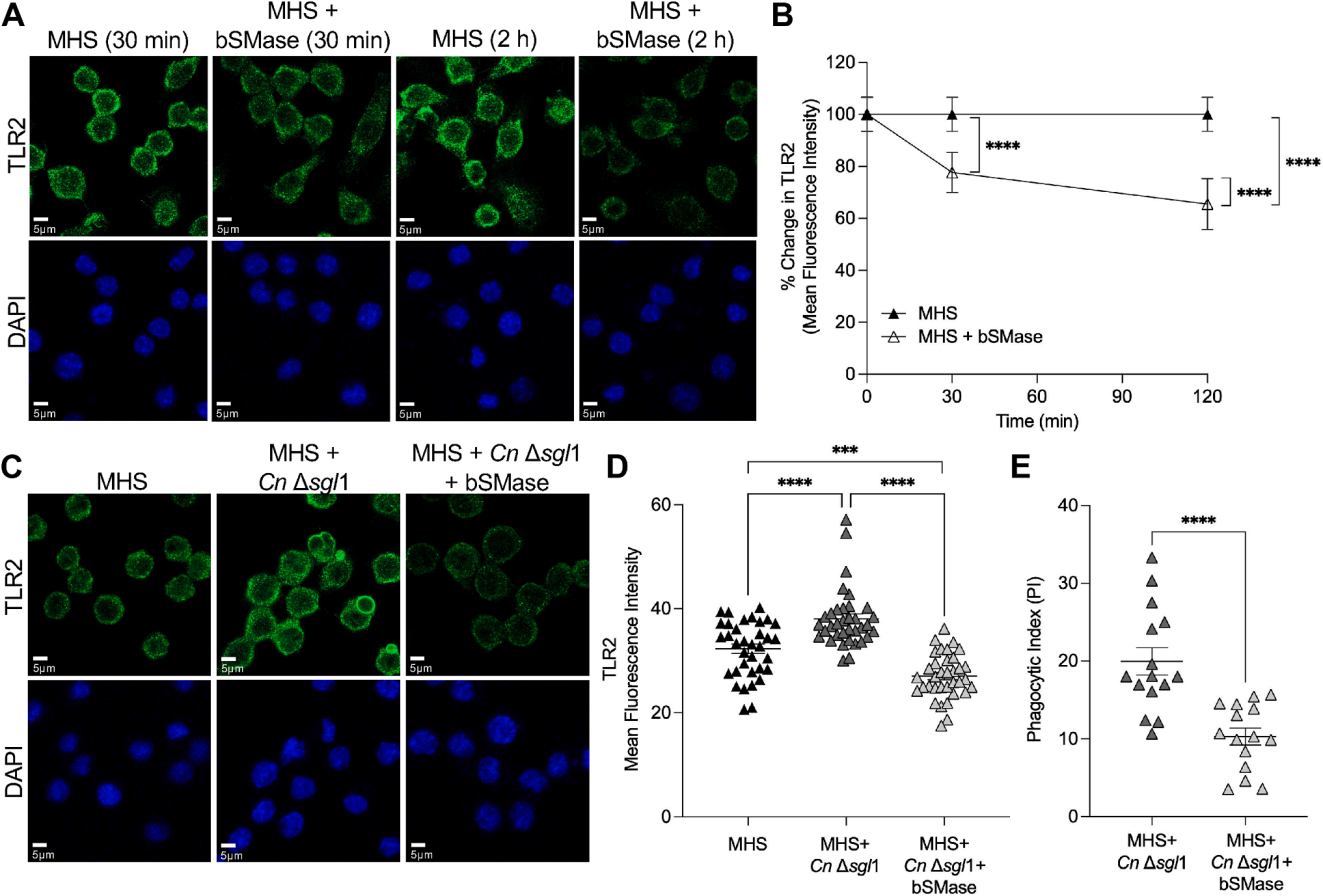
**Effect of bSMase-induced SM/ceramide imbalance on TLR2 surface localization and phagocytosis and phagocytosis in MHS macrophages.***A*, representative confocal images of the murine alveolar macrophage cell line MHS treated with bSMase for the indicated times (0, 30 min or 2 h). Plasma membrane TLR2 signal is shown in *green*, and nuclei are counterstained with DAPI (*blue*). Treatment with bSMase reduces TLR2 expression compared to untreated MHS cells. Scale bar = 5 μm. *B*, time-course quantitative analysis of plasma membrane TLR2 fluorescence shown in A at 0, 30 min, and 2 h in MHS cells with or without bSMase, expressed as percentage change relative to untreated controls. bSMase treatment resulted in significant reduction in TLR2 surface signal at 30 min and 2 h. Each *data point* represents the average of 30 MHS cells from two technical replicates in a single experiment. ∗∗∗∗*p* < 0.0001, determined by two-way ANOVA with Tukey’s multiple comparisons test. *C*, representative confocal images of treatment of MHS macrophages with bSMase decreases TLR2 activation by *Cn* Δ*sgl*1. Scale bar = 5 μm. *D*, quantitative analysis of plasma membrane TLR2 fluorescence in MHS cells infected with *Cn* Δ*sgl*1 with or without bSMase treatment. Coincubation with *Cn* Δ*sgl*1 significantly increases TLR2 surface signal compared to untreated MHS cells, and co-treatment with bSMase abrogates TLR2 expression stimulated by *Cn* Δ*sgl*1. Each *data point* represents one field of view from 3 technical replicates within a single experiment. Data are presented as mean ± SD. ∗∗∗*p* < 0.001; ∗∗∗∗*p* < 0.0001, by one-way ANOVA with Tukey’s multiple comparisons test. *E*, phagocytic index of MHS cells under the indicated treatment conditions, showing that phagocytosis of *Cn* Δ*sgl*1 is reduced by treatment with bSMase. Each *data point* represents a single field of view. At least 15 fields were acquired from 3 independent wells per condition. Data are presented as mean ± SD. ∗∗∗∗*p* < 0.0001, by one-way ANOVA with Tukey’s multiple comparisons test. TLR2, toll-like receptor 2.

We first investigated the time-dependent effect of bacterial sphingomyelinase (bSMase) on the fluorescence intensity of TLR2 at the surface of MHS cells and found that a 2-h incubation with 500 mU/ml significantly reduced TLR2 signal (∗∗∗∗*p* < 0.0001); (Fig. 6, *A* and *B*). Next, to validate the results obtained with the γδ-T cells, we assessed whether incubation with Δ*sgl*1 would increase TLR2 fluorescent signal at the plasma membrane of MHS. As shown in Figure 6, *C* and *D*, MHS cells on incubation with Δ*sgl*1 cells multiplicity of infection (MOI 1:2) for 2 h exhibited a significant increase in TLR2 fluorescence at the surface of MHS in comparison to control (∗∗∗∗*p* < 0.0001). Interestingly, treatment with bSMase completely abrogated Δ*sgl*1-induced increase in TLR2 fluorescence (Fig. 6, *C* and *D*).

To confirm that SM depletion affects phagocytosis, as previously observed (18), we tested whether bSMAse treatment of MHS would decrease phagocytosis of *C. neoformans* Δ*sgl*1. We found that depletion of SM significantly decreases the phagocytic index of MHS cells (∗∗∗∗*p* < 0.0001) (Figs. 6*E* and S1, *A* and *B*). Validation of this phenotype suggests that both the bSMAse concentration and incubation time are physiologically relevant. These results also indicate that the SM-dependent regulation of TLR2 may not be limited to γδ-T cells, but may also occur in macrophages and likely other immune cell types.

### SMS2 deficiency impairs TLR2 localization at the plasma membrane in alveolar macrophages

**Figure 7:**
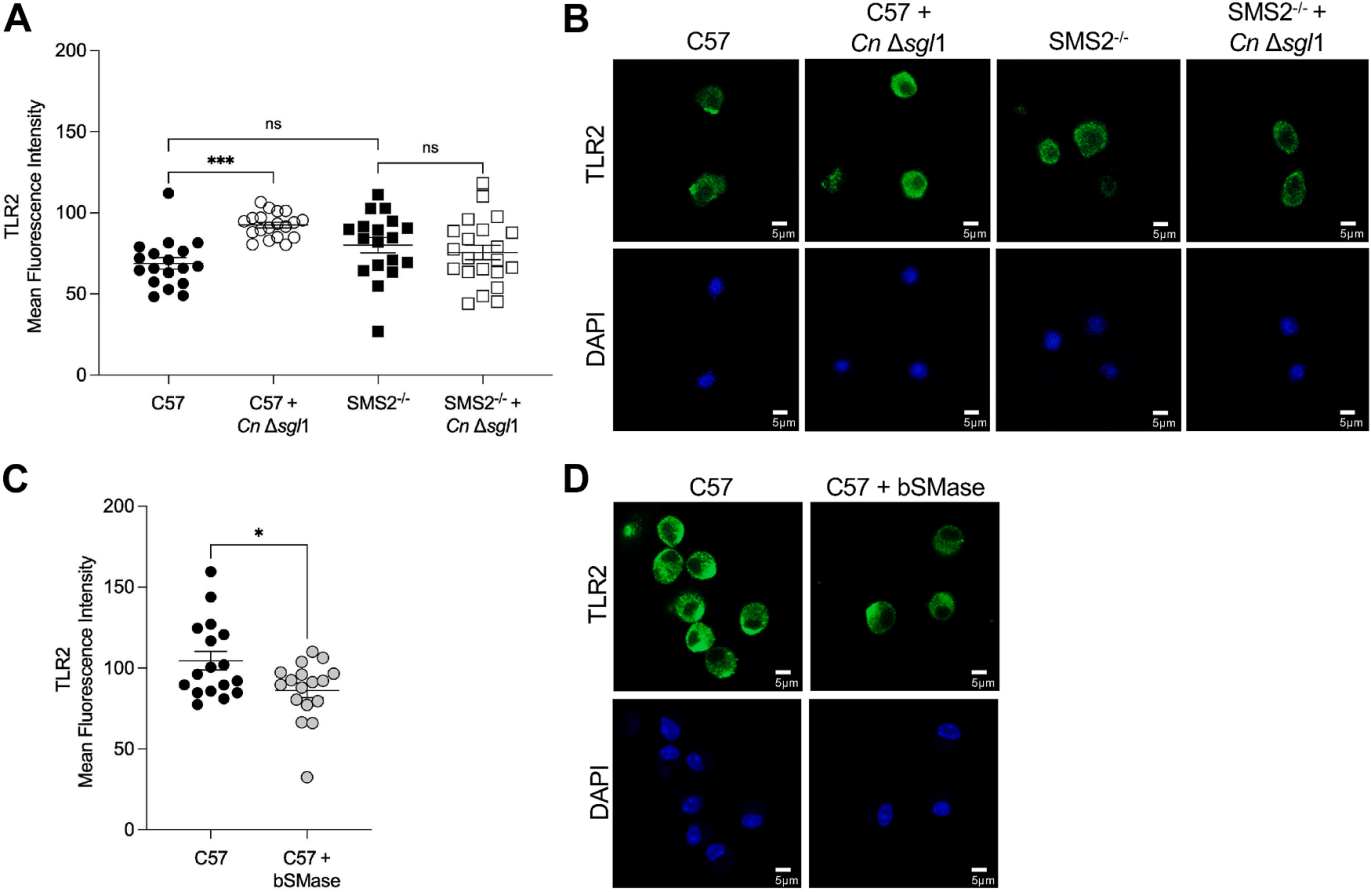
**SMS2 deficiency–associated alterations in SM/CER balance are linked to reduced TLR2 surface localization in macrophages.***A*, MFI of TLR2 in alveolar macrophages solated from WT C57BL/6 (C57) and SMS2^−/−^ mice, with or without treatment with *Cryptococcus neoformans* Δ*sgl*1 (*Cn* Δ*sgl*1) for 2 h (MOI 1:1). Macrophages were obtained from five animals per group. Each *dot* in the graph represents an individual macrophage (n = 18–21). *B*, representative immunofluorescence images showing TLR2 (*green*) and nuclei (DAPI, *blue*) in C57 and SMS2^−/−^ macrophages under the indicated conditions. The scale bar represents = 5 μm. *C*, quantification of TLR2 MFI in AMs isolated from C57 mice, with or without treatment with bSMase; 500 mU/ml for 1 h. Macrophages were obtained from five animals per group. Each *dot* represents an individual macrophage (n = 17). *D*, representative immunofluorescence images of TLR2 (*green*) and nuclei (DAPI, *blue*) in C57 macrophages ± bSMase treatment. The scale bar represents = 5 μm. Data are presented as mean ± SD. Statistical significance was determined by one-way ANOVA with Tukey’s multiple comparisons test (∗∗∗*p* < 0.001; ∗∗∗∗*p* < 0.0001). A paired Welch’s *t* test was applied where appropriate (∗*p* < 0.05; ns, non-significant). CER, ceramide; MFI, Mean Fluorescence Intensity; SMS2, sphingomyelin synthase 2; TLR2, toll-like receptor 2.

Since MHS cells showed a significant reduction in TLR2 intensity following bSMAse treatment, we next conducted an *ex vivo* study using isolated alveolar macrophages (AMs). We investigated TLR2-mediated signaling responses in AMs from C57BL/6 and SMS2^−/−^ mice upon exposure to Δ*sgl*1. We observed a significant increase in TLR2 fluorescence at the plasma membrane of AMs from C57BL/6 mice incubated with Δ*sgl*1 (multiplicity of infection, MOI, 1:1) for 2 h, compared with C57BL/6 control alone (Fig. 7, *A* and *B*). In contrast, TLR2 fluorescence in AMs from SMS2^−/−^ mice incubated with Δ*sgl*1 (MOI 1:1) remains unchanged, similar to the SMS2^−/−^ control mice as well as to C57BL/6 mice (Fig. 7, *A* and *B*).

To further examine the association between SMS2-dependent lipid changes and TLR2 surface localization in AMs, we depleted SM using bSMAse (500mU/ml) and incubated AMs isolated from C57BL/6 mice for 1 h. This resulted in a significant reduction in TLR2 fluorescence intensity compared with untreated controls (Fig. 7, *C* and *D*), further supporting the association between SMS2-dependent alterations in SM/ceramide balance and impaired TLR2 surface localization in macrophages.

### bSMase treatment alters SM/ceramide balance and is associated with reduced TLR2 membrane localization and lipid raft integrity in MHS macrophages

Because bSMase catalyzes the hydrolysis of sphingomyelin into ceramide and phosphorylcholine, its treatment simultaneously reduces sphingomyelin and increases ceramide at the plasma membrane, altering the overall lipid composition. Lipid rafts are dynamic membrane microdomains enriched in sphingolipids and cholesterol that regulate specialized cellular functions (23). TLR2 has been shown to be recruited to lipid raft clusters within these microdomains upon stimulation, facilitating downstream signaling (19, 29). Given that bSMase-driven changes in membrane lipid composition could impact raft organization, we next assessed whether these alterations are associated with changes in lipid raft integrity and TLR2 surface localization.

**Figure 8:**
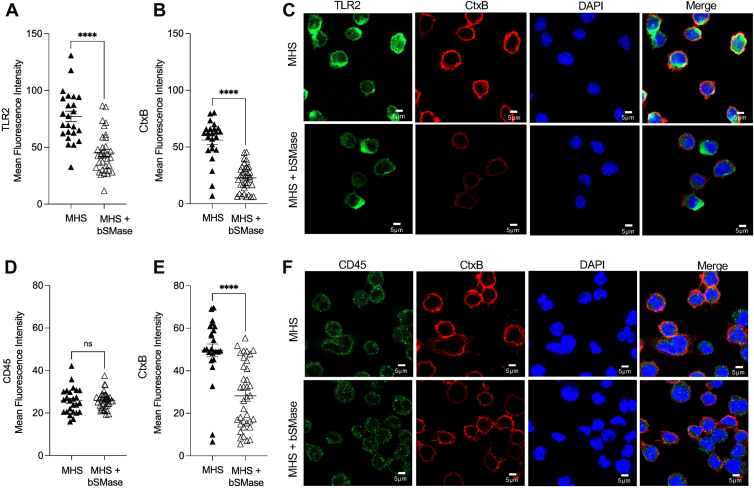
**bSMase treatment alters SM/CER balance and is associated with reduced plasma membrane TLR2 and GM1-enriched lipid raft fluorescence without affecting CD45 expression in MHS macrophages.***A*, quantification of plasma membrane TLR2 MFI in untreated and bSMase-treated MHS macrophages. *B*, quantification of GM1-enriched lipid rafts by CtxB fluorescence. *C*, representative confocal images of TLR2 (*green*), CtxB (*red*), DAPI (*blue*), and merged images in untreated and bSMase-treated cells. *D*, quantification of CD45 MFI following bSMase treatment. *E*, quantification of CtxB fluorescence corresponding to the images shown in *panel F*. *F*, representative confocal images of CD45 (*green*), CtxB (*red*), DAPI (*blue*), and merged images. bSMase-mediated sphingomyelin depletion significantly reduced plasma membrane TLR2 and GM1 (CtxB) fluorescence, whereas CD45 expression remained unchanged. Each *dot* represents an individual MHS cell from a single experiment, using n = 4 micrographs per condition. The scale bar represents = 5 μm. Data are presented as mean ± SD, with each symbol representing an individual cell. Statistical significance was determined using paired Welch’s *t* test where appropriate (∗∗∗∗*p* < 0.0001; ns, not significant). CER, ceramide; CTxB, cholera toxin B; MFI, mean fluorescence intensity; TLR2, toll-like receptor 2.

Here, we used CtxB subunit, the membrane-binding component of cholera toxin, as a well-established marker of lipid raft domains and a widely used probe for studying raft-associated membrane organization (30). Consistent with lipid raft disruption, MHS macrophages treated with bSMase, 500 mU/ml showed a significant (∗∗∗∗*p* < 0.0001) reduction in both TLR2 and CtxB fluorescence intensity compared with untreated controls (Fig. 8, *A*–*C*), whereas CD45 surface expression remains unaltered (Fig. 8, *D*–*F*). Similar results were observed in a study in which a KO of SPTLC2, a core subunit of Serine palmitoyl-CoA transferaseinvolved *de novo* sphingolipid biosynthesis, in DC2.4 cells showed significantly reduced staining for CtxB and PRRs including TLR2 whereas, CD45 expression remains unaffected, consistent with the concept that disruption of sphingolipid biosynthesis is associated with impaired lipid raft organization and reduced surface localization of raft-associated receptors including TLR2.

## Discussion

Plasma membrane lipids are increasingly recognized as active regulators of immune signaling rather than passive structural components. In particular, sphingomyelin-rich microdomains organize receptor clustering signal initiation, and spatial coordination of immune responses. However, how enzymatic regulation of sphingomyelin synthesis integrates membrane architecture with functional antifungal immunity remains poorly defined. Here, we propose that sphingomyelin synthase 2 (SMS2) acts not merely as a lipid-metabolizing enzyme but also as a spatial organizer of immune receptor accessibility, thereby linking membrane composition to vaccine-induced protection against *C. neoformans*.

SM is a major component of the animal plasma membrane, predominantly enriched in the outer leaflet. It is synthesized from phosphatidylcholine and ceramide by sphingomyelin synthase (SMS) and represents a key component of lipid rafts (12, 31). SMS1 and SMS2 are the two SMS isoforms primarily responsible for SM synthesis. SMS1 is predominantly localized to the trans-Golgi apparatus, whereas SMS2 is mainly found at the plasma membrane. SMS activity represents a rate-limiting step in SM cluster formation (32). Our analysis of bronchoalveolar lavage, lung tissue, and serum from SMS2-deficient mice showed decreased SMS activity, resulting in reduced SM synthesis, decreased relative SM abundance, and increased ceramide levels, consistent with previous reports (21, 22, 33, 34).

Functionally, SMS2 deficiency resulted in markedly delayed clearance of *C. neoformans* Δ*sgl*1 from the lungs, extending from 14 days in WT mice to 57 days in SMS2^−/−^ mice. The eventual clearance of the fungus suggests a compensatory contribution from SMS1. However, despite clearance of the Δ*sgl*1 strain, SMS2-deficient animals succumbed to secondary infection with WT *C. neoformans* H99, exhibiting increased fungal burden in both lung and brain. This occurred even after complete clearance of the Δ*sgl*1 strain, indicating that SMS2 is critical for Δ*sgl*1-mediated protective immunity.

Previous studies have linked *Δsgl*1-induced protection to γδ-T cell function as in condition of CD4^+^ or CD8^+^ T cell depletion Δ*sgl*1 still able to offer full protection in vaccinated mice, particularly through production of IFN-γ and IL-17A, which are required for full host protection in vaccinated mice (10). However, this protective response was abolished in SMS2-deficient mice immunized with either live or heat-killed Δ*sgl*1, as these animals exhibited significantly reduced IFN-γ and IL-17A production.

The mechanistic rationale for focusing on γδ-T cells in this study derives from previous work demonstrating that Δ*sgl*1-mediated vaccine protection is fully maintained under conditions of CD4^+^ or CD8^+^ T-cell depletion, strongly supporting γδ-T cells as the primary protective effector population (10). Whether conventional αβ-T cells contribute to immune regulation in immunocompetent hosts remains an open question. It is possible that in the absence of depletion, CD4+ T cells provide costimulatory or memory-forming functions that complement γδ-T cell activity. Additionally, whether TLR2 expression and its SMS2-dependent membrane localization extend to αβ-T cell subsets remains to be determined. Future studies examining TLR2 expression across T-cell subsets in SMS2-deficient mice will provide a more complete picture of how sphingomyelin-dependent membrane organization shapes the broader adaptive immune landscape during antifungal protection.

γδ-T cells are known to respond to diverse stimuli in coordination with TLRs, which enhance cytokine and chemokine production and play critical roles in early immune responses (35, 36, 37). γδ-T cells isolated from SMS2-deficient mice failed to mount robust, time-dependent production of IL-17A and IFN-γ in response to both live and heat-killed Δ*sgl*1, in contrast to cells from WT C57BL/6 mice. This defect appears to be linked to impaired TLR2 expression.

Mechanistically, *Δsgl1*-mediated vaccine protection requires TLR2 signaling and γδ-T cell-derived IL-17A and IFN-γ (10). Recent studies further demonstrate that interaction with commensal organisms activates TLR2 signaling in both dendritic cells and γδ-T cells, inducing IL-17A production. This process requires both extrinsic (dendritic cell–mediated) and intrinsic TLR2 signaling for optimal cytokine induction. Additionally, TLR2 signaling promotes epigenetic remodeling and metabolic reprogramming toward fatty acid oxidation, supporting Il17*a* transcription (38).

In this study, SMS2 deficiency was associated with a marked reduction in TLR2 surface localization on γδ-T cells following exposure to *C. neoformans* Δ*sgl*1. Given that SMS2 is a key enzyme in plasma membrane sphingomyelin biosynthesis and a critical regulator of lipid raft domains that serve as platforms for receptor organization and signaling (32), these findings suggest that SMS2-dependent alterations in the SM/ceramide balance are associated with impaired TLR2 membrane localization, which may contribute to reduced IL-17A production in SMS2-deficient γδ-T cells. Future studies using ceramide-neutralizing approaches or reconstitution with exogenous sphingomyelin will be required to dissect the individual contributions of sphingomyelin loss and ceramide accumulation to TLR2 surface localization. Furthermore, while the pattern of TLR2 signal in our imaging data is consistent with a localization defect, immunofluorescence intensity alone cannot definitively distinguish between impaired plasma membrane targeting and reduced overall TLR2 protein expression. Future studies employing qRT-PCR and flow cytometry will help resolve whether SMS2 regulates TLR2 at the transcriptional, translational, or trafficking level.

Similar defects were observed in alveolar macrophages and MHS cells, where enzymatic depletion of sphingomyelin using bSMase significantly impaired phagocytosis, an actin-dependent process in which sphingolipids contribute to pseudopod formation by promoting membrane curvature at the site of particle engulfment (23). Together, these findings suggest a broader defect in innate immune cell function in the absence of SMS2.

Overall, SMS2 deficiency alters plasma membrane lipid composition, reducing sphingomyelin and increasing ceramide, and is associated with disruption of lipid raft organization and impaired membrane domain stability and signaling function (23). This imbalance is expected to impair membrane organization and the assembly of signaling complexes. Consistently, SMS2 depletion has been shown to impair recruitment of TNFR1 upon TNF-α stimulation and to attenuate LPS-induced localization of the TLR4–MD-2 complex to the plasma membrane in macrophages (39).

Together, these findings support a unified mechanistic model in which SMS2 maintains sphingomyelin-enriched membrane domains that spatially organize immune receptor function. In this model, SMS2-dependent lipid architecture regulates TLR2 localization and clustering at the plasma membrane, enabling γδ-T cell cytokine responses (IL-17A and IFN-γ), macrophage phagocytic function, and effective fungal clearance. Loss of SMS2 disrupts sphingomyelin homeostasis and lipid raft integrity, leading to impaired receptor organization, defective immune signaling, and failure of vaccine-induced protection against *C. neoformans*.

Overall, this work reframes SMS2 as a spatial regulator of immune competence rather than a conventional metabolic enzyme, highlighting membrane sphingomyelin architecture as an important determinant of effective antifungal vaccination. By organizing sphingomyelin-enriched membrane domains, SMS2 contributes to the spatial coordination of immune receptor function, including the TLR2-dependent signaling axis previously shown to be essential for Δ*sgl*1-mediated protection (10). These findings support a model in which membrane lipid composition sets the threshold for coordinated innate and innate-like immune responses required for durable antifungal immunity.

## Experimental procedure

### Cell culture condition

The murine alveolar macrophage cell line MH-S (American Type Culture Collection, CRL-2019) was maintained in RPMI 1640 medium supplemented with 10% heat-inactivated fetal bovine serum and 1% penicillin–streptomycin and cultured at 37 °C in a humidified atmosphere containing 5% CO_2_. The cell line was authenticated by ATCC and routinely tested to confirm the absence of *mycoplasma* contamination. For experimental procedures, cells were seeded at densities appropriate to each assay, such as 5 × 10^4^ cells per well in a final volume of 200 μl in 96-well plates, 1 × 10^5^ cells per well in 350 μl in an 8-chamber slide. Following seeding, cells were allowed to adhere overnight under standard culture conditions before experimental treatments.

### *Cryptococcus* strains

Two strains of *C. neoformans* were used in this study: the WT *C. neoformans* var. *grubii* strain H99 serotype A (WT), and *C. neoformans* Δ*sgl*1, a mutant strain accumulating sterylglucosides (7). These fungal strains were recovered from glycerol stock stored at −80 °C and streaked on YPD plates, incubated at 30 °C for 48 to 72 h. An isolated colony was picked and added to 10 ml of YPD broth and grown at 30 °C for 16 to 18 h with shaking. Next day, cells were washed with sterile PBS, counted on a hemocytometer, and resuspended in sterile PBS at the desired concentration. For heat-killed strains, yeast cultures were adjusted to the required concentration in PBS and incubated at 80 °C for 75 min. To confirm cell death, aliquots were plated onto the YPD plates and plates were incubated at 30 °C for up to 4 days. Absence of any colony indicates successful killing.

### Animal housing conditions and ethical statement

Male C57BL/6J mice (strain #000664) (5–7 weeks old) were purchased from The Jackson Laboratory and housed in the Division of Laboratory Animal Resources (DLAR) facility at Stony Brook University. 3 to 4 animals were housed per cage under specific pathogen-free conditions and were acclimatized for 1 week before the initiation of experiments provided food pellets and water *ad libitum*. A Breeding colony of the SMS2 full KO (SMS2^−/−^) mice were obtained from Dr Xian C. Jiang and maintained at Stony Brook through PCR-based genotyping. Only confirmed SMS2^−/−^ mice were maintained under standard housing conditions and used for the experiments. All animal procedures were approved by the Stony Brook University Institutional Animal Care and Use Committee (protocol no. 341588), and experiments were conducted in accordance with protocols approved by the Institutional Animal Care and Use Committee (IACUC).

### Lipid analysis from lung, BAL, and serum

Animals were euthanized by CO_2_ asphyxiation, and lung tissue was collected from C57BL/6 or SMS2^−/−^ mice and stored at −80 °C until sample processing. At the time of sample preparation for lipid analysis, the sample was thawed on ice, and homogenized using an auto-homogenizer in homogenization buffer (20 mM Tris pH 7.8 with protease and phosphatase inhibitors), and centrifuged for 5 min at 300*g* and stored at −80 °C until lipid analysis.

BAL was collected from C57BL/6 and SMS2^−/−^ mice euthanized by CO_2_ asphyxiation, then a small nick was created, and the trachea was exposed. Using 20*g* Luer adapter tightened with silk thread, 1 ml syringe containing PBS was connected to collect BAL fluid, collecting at least 5 ml of BAL fluid per sample. Centrifuged to collect the cell pellet and stored at −80 °C until lipid analysis.

For serum collection, animals were euthanized by CO_2_ asphyxiation, and whole blood was collected in BD tubes and left undisturbed at RT for 15 to 30 min. Later centrifuged at 2000*g* for 10 min at 4 °C, and the resulting supernatants were designated as serum and stored to −80 °C until lipid analysis.

For sphingomyelin (SM) and ceramide species, the methods used were as per (40). Briefly, lipid was extracted from the BAL, lung, and serum samples collected from C57BL/6 and SMS2^−/−^ mice (n = 3 mice per group per time point) in 2 ml isopropanol: water: ethyl acetate (30:10:60 by volume) and analyzed by reverse-phase high-pressure LC coupled to electrospray ionization and subsequent separation by MS. Analysis of SM and ceramide species was performed on a TSQ Quantum Ultra Mass Spectrometer (Thermo Fisher Scientific), operating in a multiple reaction monitoring positive ionization mode. Sphingolipid levels were normalized to the inorganic phosphate concentration measured in the original cell extract.

### Animal infection, survival, and fungal burden

Animals were grouped according to the experimental conditions (3–4 in each group for fungal burden studies or 10 in each group for survival studies) and acclimated for a week before cryptococcal infection. On the day of the infection, mice were anesthetized using an intraperitoneal injection (i.p.) of a 40 μl cocktail of xylazine-ketamine, containing 95 mg of ketamine per kilogram of body weight and 5 mg of xylazine per kilogram of body weight. These anesthetized C57BL/6 (C57) WT mice were immunized with 5 × 10^5^ cells of *C. neoformans* Δ*sgl*1 suspended in 20 μl PBS, while another group of C57 mice received 20 μl sterile PBS (as control). Similarly, SMS2^−/−^ mice also received 5 × 10^5^ cells of *C. neoformans* Δ*sgl*1suspended in 20 μl PBS, while another group of SMS2^−/−^ mice received 20 μl sterile PBS (as control). As per the experimental plan, after either 30 days or an extended 75 days post-immunization, mice were challenged intranasally with 5 × 10^5^ cells of WT *C. neoformans* in 20 μl PBS. Mice were observed daily and if they display any signs of severe disease—including laboured breathing, neurological symptoms, or a body weight loss greater than 20% —were humanely euthanized by CO_2_ asphyxiation. For tissue burden studies, organs, such as brain, and lungs, were collected, either at the time-of-death (euthanasia) of at the end of the experiment. Organs were then homogenized in 10 ml sterile PBS using a Stomacher 80 blender and serial dilution plated onto a YPD agar. Plates were incubated at 30 °C for 3 days, and colony forming units (CFUs) were counted to determine organ fungal burden.

### Splenocytes isolation, stimulation, and cytokine release estimation through ELISA

Mice were euthanized by CO_2_ asphyxiation, and spleens were harvested from C57BL/6 and SMS2^−/−^ mice. Single-cell suspension was prepared in MACS buffer and centrifuged at 400*g* for 5 min. Red blood cells were lysed using ACK lysis buffer, followed by washing with MACS buffer. γδ T cells were isolated from the splenocytes in 100 μl complete RPMI medium using a mouse γδ-T cell isolation MACS separation kit (Miltenyi Biotec, catalog no. 130-092-125) using LD and MS columns, according to the manufacturer’s instructions.

A total of 1 × 10^5^ live γδ T cells were seeded in complete RPMI medium and infected with *C. neoformans* WT or *C. neoformans* Δ*sgl*1strains at a MOI of 1:2. Cells were incubated at 37 °C with 5% CO_2_ for 5 days. Supernatants were collected at 1, 3, and 5 days post-infection and stored at −80 °C until cytokine analysis. The levels of IFN-γ and IL-17A in the supernatants were measured using Legend Max Mouse IFN-γ and IL-17A ELISA kits (BioLegend), following the manufacturer’s instructions.

### Alveolar macrophage isolation and culture conditions

Alveolar macrophages were isolated by bronchoalveolar lavage as described with slight modification (41). Briefly, mice were euthanized by CO_2_ asphyxiation, and bronchoalveolar lavage was performed by flushing the lungs with PBS using an intratracheal catheter and a 1 ml sterile syringe, with a total of 5 to 6 ml instilled and recovered per mouse. The collected lavage fluid was centrifuged at 300*g* for 10 min at 4 °C, and the resulting cell pellet was resuspended in alveolar macrophage (AM) culture medium consisting of RPMI 1640 supplemented with 10% heat-inactivated FBS, 1 mM sodium pyruvate, 10 mM HEPES, and 1% penicillin–streptomycin. After 2 h of incubation, nonadherent cells were washed off with PBS, and the medium was refreshed.

### Sphingomyelin depletion using bSMase

To selectively deplete sphingomyelin (SM) from the outer leaflet of the plasma membrane, macrophages were treated with bSMase, which catalyzes the hydrolysis of SM into ceramide and phosphorylcholine, as previously described (18, 42). Briefly, Macrophages were seeded at the appropriate density and allowed to adhere overnight in a humidified incubator maintained at 37 °C with 5% CO_2_ to establish a stable monolayer. Before treatment, macrophage monolayers were washed twice with sterile 1 × Dulbecco’s PBS to remove residual serum components that could interfere with enzyme activity. Freshly prepared bSMase was added to serum-free culture medium at a final concentration of 500 mU/ml at appropriate volumes for the culture format, 8-well chamber slides, and incubated for different times 0, 30 min, and 2 h at 37 °C in a humidified atmosphere containing 5% CO_2_, later expression of TLR2 was measured through immunofluorescence.

### Phagocytosis assay and giemsa staining

MHS cells were seeded at a density of 5 × 10^4^ cells per well in 96-well tissue culture plates and at 1 × 10^5^ cells per chamber of 8 chamber slide, with a volume of 200 and 350 μl DMEM, respectively, supplemented with 10% heat inactivated fetal bovine serum and 1% penicillin–streptomycin (P/S). Cells were incubated overnight at 37 °C in a humidified incubator with 5% CO_2_. The phagocytosis assay was performed following the protocol described by Bryan *et al*. (43).

Briefly, *C. neoformans* strains H99 or *C. neoformans* Δ*sgl*1 were cultured by inoculating a single colony from a streaked agar plate into 10 ml yeast nitrogen base medium and incubating overnight at 30 °C with shaking. The next day, cells were harvested by centrifugation at 1700*g* for 10 min at 4 °C, washed three times with 5 ml of 1 × PBS, and resuspended in 1 × PBS. Fungal cells were counted and adjusted to achieve a MOI of 1:2 (MHS cells: *C. neoformans* strains). The fungal cells were then opsonized using 1 μg/ml anti-GXM antibody per 10^5^ *C. neoformans* Δ*sgl*1cells in complete medium (DMEM containing 10% FBS and 1% P/S) and added to macrophage-like cells activated with 50 units/ml of recombinant murine gamma interferon and 0.3 μg/ml of lipopolysaccharide and total volume was adjusted to plating conditions per well using DMEM supplemented with 10% FBS and 1% P/S, vortexed briefly, and incubated for 20 min at 37 °C, later this cocktail was added to the MHS cells, incubated for 2 h. Following 2 h of incubation MHS monolayer was air-dried for approximately 10 min and fixed with 200 μl ice-cold methanol for 15 min at RT. After fixation, methanol was removed, and cells were stained with 200 μl Giemsa stain for 5 min at RT. Wells were washed 2 to 3 times with deionized water to remove excess stain and allowed to air-dry overnight at RT with the plate lid removed. For sphingomyelin depletion, 500 mU/ml bSMase (Sigma #567707) was added to the opsonized medium containing *C. neoformans* cells, IFN-γ, and LPS immediately before infection, followed by incubation at 37 °C with 5% CO_2_ for 2 h. Giemsa staining was then performed to determine the phagocytic index, and images were acquired using a Zeiss light microscope for higher magnification at Nikon microscope. Phagocytic index is calculated as the percentage of yeast cells ingested per number of macrophages per field. For each experiment, at least 15 fields were observed in at least 3 different wells per condition.

### Immunofluorescence

MHS cells were seeded at 1 × 10^5^ cells per chamber of 8 chamber slide, media supplemented with 10% heat inactivated fetal bovine serum and 1% penicillin–streptomycin (P/S). Cells were incubated overnight at 37 °C in a humidified incubator with 5% CO_2_, next day cells were infected with *C*. *neoformans* Δ*sgl*1 (MOI 1:2) opsonized with anti-GXM in appropriate media volume containing LPS and IFN_ϒ_ as mentioned previously and incubated for 2 h, later cells were washed with PBS and performed immunofluorescence following a protocol (44) and modified slightly as per the experimental study. Briefly, after PBS wash cells were fixed for 15 to 20 min with 4% paraformaldehyde at RT, then blocked for 30 min with superblock (Scytek # AAA999) containing 0.02% Triton X, incubated overnight in primary antibody TLR2 (Invitrogen #MA5-16260) in (1:100) at 4 °C in humidified chamber, next day washed thrice in wash buffer consisting super block in PBST, incubated secondary antibody (Invitrogen #A11006) dilution (1:1000) for 30 to 45 min at RT washed again and mounted using ProLong Gold Antifade Mountant (invitrogen #P36935). For TLR2 expression in γδ T cells 1 × 10^5^ live γδ-T cells were seeded per well in 350 μl in 8-chamber slide under standard culture conditions 37 °C with 5% CO_2_, and incubated with *C. neoformans* Δ*sgl*1 (MOI 1:2) opsonized with anti-GXM in appropriate media volume containing LPS and IFN_ϒ_ as mentioned previously and incubated for 2 h, later cells were washed with PBS and fixed for 15 to 20 min with 4% paraformaldehyde at RT, and immunofluorescence was performed as described. Later, cells were imaged using a confocal microscope (Zeiss LSM 980) to assess TLR2 expression, and the mean fluorescent intensity was quantified using ImageJ (https://imagej.net/ij/). For CtxB labelling as described (23) and was slightly modified, MHS cells were washed with Cold PBS and incubated on ice with CtxB (Invitrogen #C34776) for 10 to 15 min, then washed quickly with PBS and fixed with 4% paraformaldehyde at RT for 10 to 15 min, later washed with PBS and blocked with BSA for 30 min, and then incubated overnight with TLR2 primary antibody and again washed with PBS and added secondary antibody at RT for 30 to 45 min mounted and performed confocal imaging.

MA5-16260 antibody was verified by the manufacturing company by cell treatment to ensure that the antibody binds to the antigen stated. Altered expression of the target protein upon cell treatment demonstrated antibody specificity. A-11006 secondary antibody is a Goat-anti-rat IgG and validation was performed by the manufacturing company.

### Statistical analysis and study design

All statistical analyses were performed using GraphPad Prism 10 software (https://www.graphpad.com/features). Sample sizes, statistical methods, and measures of significance are detailed in the corresponding figure captions. All collected data were included in the analyses without exclusion. *In vivo* survival studies were performed using 10 mice per group to achieve sufficient statistical power while minimizing animal use. Survival differences were evaluated using the log-rank (Mantel–Cox) test. For comparisons between two groups, an unpaired *t* test was used; for comparisons among more than two groups, statistical significance was determined by one-way ANOVA with Tukey’s multiple comparisons test. Data are presented as mean ± SD

### Data availability

All data are contained within the article.

## Supporting information

This article contains supporting information.

## Conflict of interest

The authors declare that they have no conflicts of interest with the contents of this article.
