# Supplementary material for: Sphingomyelin synthase 2 regulates host protection conferred by the administration of Cryptococcus neoformans Δsgl1 strain
Source: J Biol Chem. 2026 Jul 13;302(9):113330. doi: 10.1016/j.jbc.2026.113330 (PMC13475412; doi:10.1016/j.jbc.2026.113330)
Supplement: Supplementary file 1 [file mmc1.pdf]

Supplementary Material

Sphingomyelin synthase 2 regulates host protection conferred by the administration of *Cryptococcus neoformans* Δ*sgl*1 strain

Neelu Singh[1], Tyler G. Normile[1], Deveney Dasilva[1], Nivea Pereira de Sa[1],

and Maurizio Del Poeta[1,2,3 #]

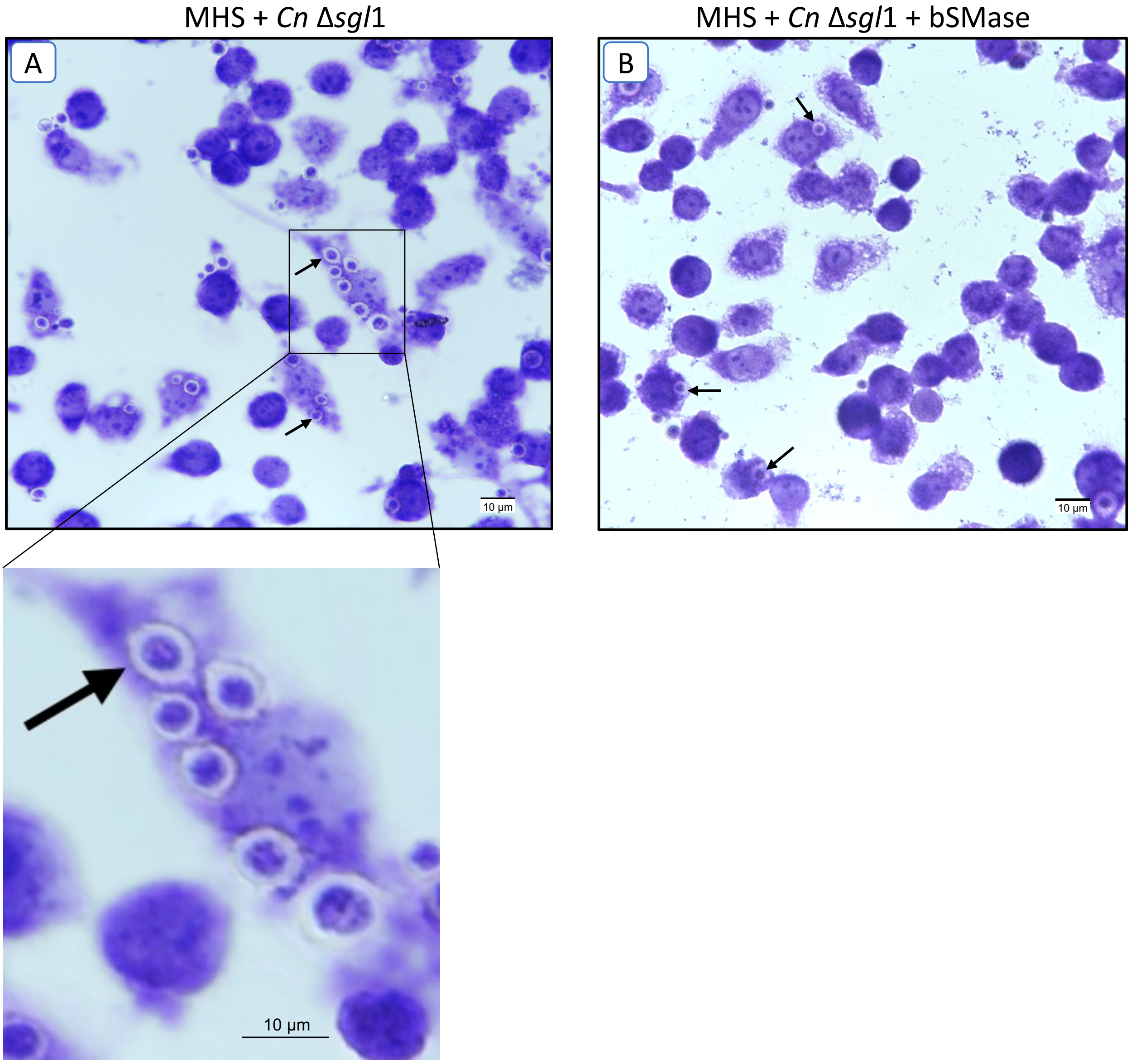

Supplementary Figure S1. Phagocytosis of *Cryptococcus neoformans* Δ*sgl1* by MHS macrophages. Representative brightfield images (100x) of MHS macrophages incubated with *Cryptococcus neoformans* Δ*sgl1* in the absence or presence of bacterial sphingomyelinase (bSMase), followed by Giemsa staining. **A)** MHS macrophage incubated with Δ*sgl1 (*MOI 1:2) for 2h. Multiple yeast cells are visibly internalized within macrophages (indicated by black arrows), where macrophages efficiently engulf the fungus. **B)** Macrophages treated with bacterial sphingomyelinase (bSMase) 500mU/mL, which depletes membrane sphingomyelin, resulting in reduced uptake of fungi, indicating impaired phagocytosis. Scale bar = 10 µm
